# Stacking sequence determines Raman intensities of observed interlayer shear modes in 2D layered materials – A general bond polarizability model

**DOI:** 10.1038/srep14565

**Published:** 2015-10-15

**Authors:** Xin Luo, Xin Lu, Chunxiao Cong, Ting Yu, Qihua Xiong, Su Ying Quek

**Affiliations:** 1 Centre for Advanced 2D Materials and Graphene Research Centre, National University of Singapore, 6 Science Drive 2, Singapore 117546; 2 Department of Physics, National University of Singapore, 2 Science Drive 3, Singapore 117551; 3 Institute of High Performance Computing, 1 Fusionopolis Way, #16-16 Connexis, Singapore 138632; 4 Division of Physics and Applied Physics, School of Physical and Mathematical Sciences, Nanyang Technological University, Singapore 637371; 5 NOVITAS, Nanoelectronics Centre of Excellence, School of Electrical and Electronic Engineering, Nanyang Technological University, Singapore 639798.

## Abstract

2D layered materials have recently attracted tremendous interest due to their fascinating properties and potential applications. The interlayer interactions are much weaker than the intralayer bonds, allowing the as-synthesized materials to exhibit different stacking sequences, leading to different physical properties. Here, we show that regardless of the space group of the 2D materials, the Raman frequencies of the interlayer shear modes observed under the typical 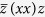 configuration blue shift for AB stacked materials, and red shift for ABC stacked materials, as the number of layers increases. Our predictions are made using an intuitive bond polarizability model which shows that stacking sequence plays a key role in determining which interlayer shear modes lead to the largest change in polarizability (Raman intensity); the modes with the largest Raman intensity determining the frequency trends. We present direct evidence for these conclusions by studying the Raman modes in few layer graphene, MoS_2_, MoSe_2_, WSe_2_ and Bi_2_Se_3_, using both first principles calculations and Raman spectroscopy. This study sheds light on the influence of stacking sequence on the Raman intensities of intrinsic interlayer modes in 2D layered materials in general, and leads to a practical way of identifying the stacking sequence in these materials.

Two dimensional (2D) layered materials have recently attracted much attention from both research and industry communities, mainly because of their unique thickness and symmetry dependent electronic and optical properties. Among these 2D layered materials, few layer graphene (FLG), 2D transition metal dichalcogenides (TMD, such as MoS_2_), and thin films of topological insulators Bi_2_X_3_ (X = Te, Se) are of particular interest due to their potential in novel applications. Dimensionality, symmetry and stacking orders play a critical role in the properties of layered materials. For example, 3-layer graphene (3LG) in Bernal (AB) stacking is a semimetal without a bandgap, while a spontaneous band gap can be opened in the rhombohedral (ABC) stacked 3LG with symmetry-breaking ground states^1^. ABA stacked three layer graphene exhibits unconventional quantum hall effects originating from the mirror symmetry with respect to the middle graphene layer^2^. Single layer MoS_2_ was recently theoretically proposed and later experimentally demonstrated to be a good candidate in valleytronic applications due to the absence of inversion symmetry and large spin-orbit coupling^3^,^4^,^5^,^6^,^7^. Unlike the more common 2H type (AB stacked) MoS_2_ with inversion symmetry, the 3R (ABC stacked) few layer MoS_2_ is also a noncentrosymmetric material, and enhanced valley polarizations are observed on 2–4L ABC stacked MoS_2_ in circularly polarized photoluminescence measurements^8^. More recently, the array of 2D materials available has become increasingly diverse, with new candidates such as black phosphorus, iron selenides, etc. coming into play^9^,^10^.

Infrared (IR) and Raman spectroscopy have become a convenient, sensitive and non-invasive procedure to characterize 2D layered materials^11^,^12^,^13^,^14^,^15^. The interlayer frequencies from Raman spectrcopy are used as a fingerprint for the number of layers in these 2D materials, and as a probe for interlayer coupling in heterostructures^16^. By using IR absorption spectroscopy, Mak^17^ and Li^18^ reported unambiguous evidence for the existence of both AB and ABC stacked FLGs. On the other hand, the Raman measurements on AB and ABC stacked FLG revealed distinct line shapes for the 2D Raman mode although their frequencies are almost the same^17^. This difference in line shape is used to spatially image the different stacking orders in FLG^19^,^20^.

While details of Raman and IR spectra can be used to identify the stacking sequence in specific materials, the widening array of newly discovered 2D materials begs the question of whether or not there are any rules of thumb that can be used to identify the stacking sequence regardless of the specific details of the 2D material. Common to all 2D layered materials are the interlayer shear and breathing modes, in which each layer moves as a whole unit^12^,^13^,^14^,^21^,^22^. Indeed, recent experiments and calculations have uncovered such modes in AB stacked FLG^18^,^22^,^23^,^24^ and TMD^12^,^14^, as well as in Bi_2_Se_3_ and Bi_2_Te_3_ (which is ABC stacked by nature)^23^. Interestingly, it was found that as the number of layers increases, the Raman frequencies of the observed interlayer shear mode blue shift in AB stacked FLG and TMD^12^,^14^,^21^, but red shift in ABC stacked Bi_2_Se_3_ and Bi_2_Te_3_^23^. The different frequency evolution trends arise from the different Raman intensities of the available interlayer shear modes. These trends cannot be predicted from group theory, because many Raman-active modes turn out to have zero Raman intensity^12^. Instead, we show that the frequency trends stem from Raman modes with the highest Raman intensity, which we can predict using a simple bond polarizability model that requires only information of the relative atomic positions and displacements. The model shows that stacking sequence plays a key role in determining which interlayer shear modes lead to the largest change in polarizability (Raman intensity). Based on this model, we show that the above-mentioned correlation between stacking sequence and frequency trends is general, and can be used to determine the stacking sequence in different materials. We present direct support for these conclusions using first principles calculations as well as Raman spectroscopy measurements.

## Results

### Interlayer modes illustrated by few layer graphene

We begin by discussing the interlayer modes present in 2D layered materials. In a 2D material with N layers, there are N times the number of normal modes as that in the monolayer. Each normal mode in 1 layer (1L) evolves into N modes with slightly different frequencies in N layers (NL), keeping the same intralayer displacement while varying the phase difference between adjacent layers^12^. The difference in frequencies arises from the difference in relative phases in adjacent layers. In this way, the interlayer modes in NL correspond to the acoustic mode in 1L, in which all atoms within the single layer move together. Since the interlayer interactions are weak, the corresponding frequencies are also low.

**Figure 1:**
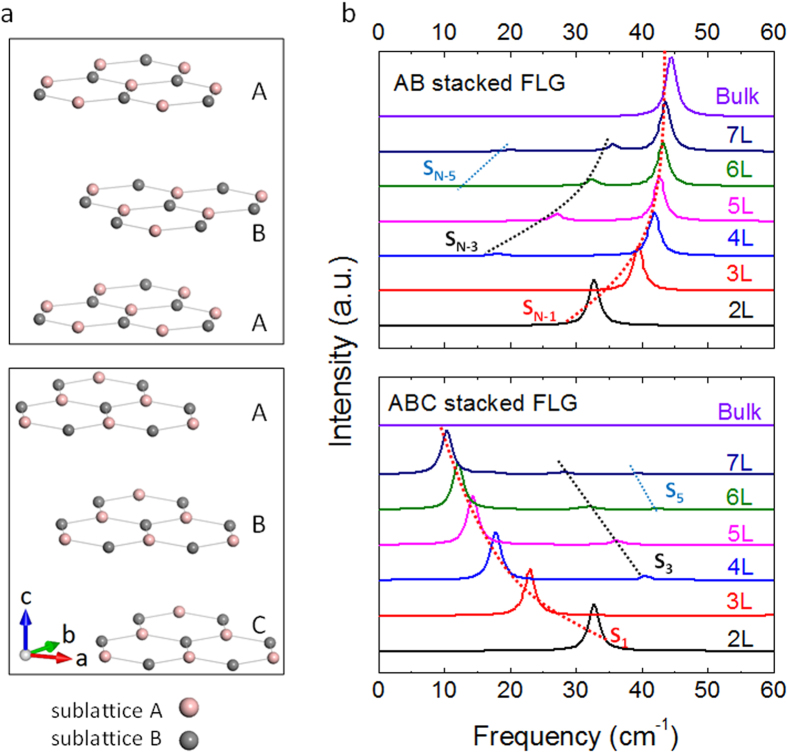
(**a**) Structural illustrations of AB and ABC stacked order in 3L graphene (extended periodically in the a and b directions). Pink and gray balls denote atoms in the two sublattices. (**b**) LDA calculated low-frequency Raman spectra in AB and ABC stacked few layer graphene (FLG) and bulk graphite. Dashed lines guide the frequency evolution trends of the interlayer shear modes.

As an illustration, we show in Fig. 1 the ultralow frequency Raman spectra of AB and ABC stacked FLG with varying thickness, computed using density functional theory (DFT) within the local density approximation (LDA) (see Methods for details). These LDA calculated frequencies are in excellent agreement with experiments (Supplementary Figure S1). We note that unless otherwise stated, the Raman intensities discussed in this work refer to the non-resonant Raman intensities. The Raman intensities for each line are normalized by the largest value in that system. Unless otherwise mentioned, we shall consider in this work the Raman intensities corresponding to those obtained in the most common 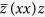 polarization configuration. We use the notation “S” to label the interlayer shear modes and the subscripts, 0 to (N-1), denote in order the lowest to highest frequency shear modes in each system, with S_0_ corresponding to the acoustic mode, S_1_ the lowest frequency shear mode and S_N-1_ the highest frequency shear mode. Supplementary Tables S1 and S2 show the frequencies of all the interlayer modes in AB and ABC stacked FLG respectively – the stacking order has negligible impact on the frequency values, indicating that the interlayer force constants are similar in both systems (see also caption of Supplementary Figure S1). However, distinct frequency trends are found in AB and ABC stacked FLG (Fig. 1b). The peaks with largest intensity correspond to S_N-1_ and S_1_ in AB and ABC stacked FLG respectively. The other trends (S_N-3_ and S_N-5_ in AB stacked FLG, and S_3_ and S_5_ in ABC stacked FLG) are barely visible in Fig. 1b, but can be seen from the details in Supplementary Tables S1 to S2.

**Figure 2:**
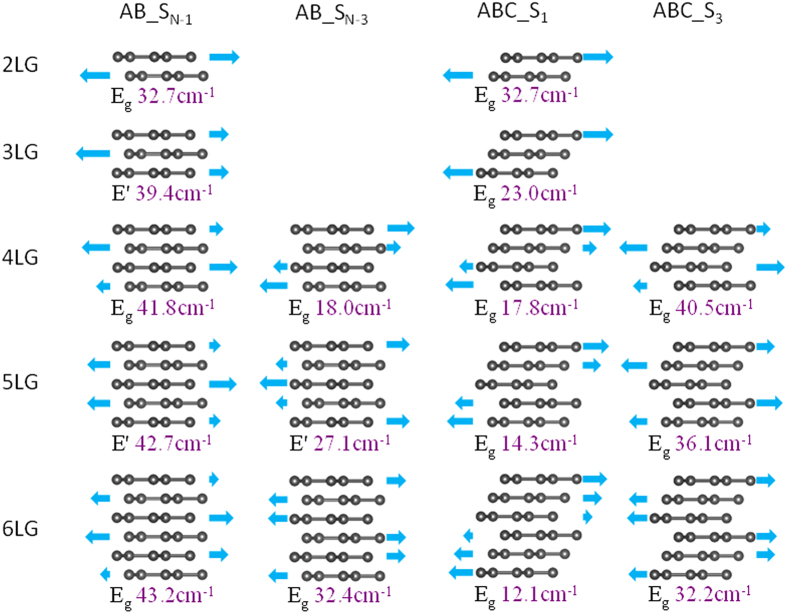
The atomic displacements and frequencies of the S_N-1_ and S_N-3_ modes in AB stacked FLG and S_1_ and S_3_ modes in ABC stacked FLG.

As we have seen, the subscript of the S mode determines its relative Raman intensity. Furthermore, this subscript, i.e. whether a mode is the lowest or highest frequency shear mode, or the third lowest or third highest frequency mode, dictates whether the frequencies red shift or blue shift with increasing thickness. In general we find that the lower-frequency shear modes red shift with increasing thickness, and the opposite is true for the higher-frequency shear modes. To understand this, we consider the atomic displacements of the S_N-1_, S_N-3_, S_1_ and S_3_ modes as shown in Fig. 2. First we note that the highest frequency mode S_N-1_ corresponds to maximally out-of-phase displacement between adjacent layers, while the lowest frequency mode S_1_ corresponds to minimum out-of-phase displacement. Since the adjacent graphene layers vibrate out of phase (180^o^) in the S_N-1_ modes, the restoring forces accumulate with increasing thickness, resulting in the blue shift with thickness. In contrast, for the S_1_ modes, the graphene layers are equally divided into two parts, with one part moving in one direction and the other part moving in the opposite direction, and the atomic displacements are gradually reduced towards the interface of the two parts. The relative displacement between adjacent layers therefore decreases as the number of layers increases, resulting in a red shift. Similar analysis can apply to the S_N-3_ and S_3_ modes.

For completeness, we note that besides interlayer shear modes, interlayer breathing modes are also present; the atomic displacements being similar to those of the shear modes, but in the out-of-plane direction. In FLG, the intensities of such modes are much smaller than the shear modes. We note that unlike the shear modes, the stacking order has no influence on the relative Raman intensities of the breathing modes (Supplementary Figure S1, Tables S1–S2).

It is interesting to note that the stacking order dependent frequency trends for FLG are consistent with the trends for AB stacked TMD and ABC stacked Bi_2_Se_3_ and Bi_2_Te_3_ that are reported in the literature^12^,^13^,^14^,^21^,^23^,^24^ – in other words, the frequency of the observed interlayer shear mode blue (red) shifts for AB (ABC) stacked materials as the number of layers increases. Just as in the case of FLG, these frequency trends are determined by which shear modes (lowest or highest frequency) have the largest Raman intensities^12^,^13^,^14^,^23^,^25^. The observation of these same trends in vastly different materials with similar stacking order begs the question of whether the stacking order (AB or ABC stacking) can in general determine the Raman intensities of the interlayer shear modes and therefore the observed frequency trends.

The Raman *activity* of a phonon mode is assigned using group theory analysis, which tells us about the symmetries present in the material. It is natural to question whether the space group (symmetries) of these 2D layered materials can also explain the stacking-order dependent Raman *intensities*. We summarize in Table 1 the space group (symmetries) of multi-layered graphene, MoS_2_ (MoSe_2_ and other 2H TMD) and Bi_2_Se_3_ (Bi_2_Te_3_) under AB and ABC stacking, with different number of layers. It is clear that the space group (symmetry) does not explain the Raman intensities for the interlayer shear modes. For example, both AB and ABC stacked graphene with an even number of layers have the same symmetry, even though the interlayer shear modes for these two systems have very different Raman intensities. Likewise, ABC stacked Bi_2_Se_3_ and Bi_2_Te_3_ have the same space group (D^3^_3d_) as AB and ABC stacked graphene with an even number of layers, but the observed frequency trends follow only those of ABC stacked graphene. On the other hand, although ABC stacked graphene (D^3^_3d_) and ABC stacked MoS_2_ and MoSe_2_ (C^1^_3v_) have different symmetry, the Raman spectra for their interlayer shear modes share the same frequency trends. Therefore, it is the stacking order that determines the Raman intensities of the interlayer shear modes. The symmetry determines the Raman activity of each mode, but not the Raman intensities.

In what follows, we present a bond polarizability model that uses information about the relative atomic coordinates and atomic displacements of the vibration modes, without explicitly considering group symmetries. This model provides an intuitive explanation for the stacking-order dependent Raman intensities and frequency trends.

### Bond Polarizability Model

In the first principles calculations, the nonresonant Raman intensity of a phonon mode *k* is computed in the Placzek approximation^26^:


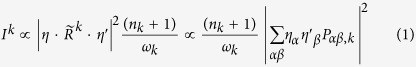


where 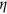 and 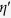 are the unit vectors for the polarization of the incident and scattered light, 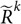 is the second rank Raman tensor, 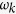 and 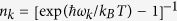 are the frequency and the Boltzmann distribution function of phonon mode *k*, respectively. 
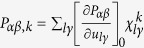
 is the derivative of the electronic polarizability tensor 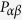 with respect to the atomic displacement. 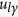 is the displacement of *l*th atom in the 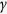 direction for normal mode *k* and 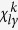 is the 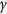
th (*x*, *y*, or *z*) compononent of the eigenvector of phonon mode *k*. Here we consider the backscattering geometry with the polarization of 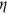 and 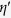 parallel to the in-plane *a* axis, i.e. 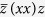 in Porto notations^27^. From equation (1), one can deduce that for the same frequency and occupation numbers, the Raman intensity is proportional to the change in polarizability of the system, when the atoms are displaced from equilibrium in the direction of the phonon eigenvector. The polarizability is defined as the induced dipole moment relative to the applied electic field. For the 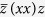 configuration, the relavant polarizability component would be the *xx* component, i.e. the dipole moment in the *x* direction, induced by an applied electric field in the *x* direction.

As the atoms are being displaced and bonds stretched/compressed, it is reasonable to consider that the major change in polarizability will arise from changes in the relevant dipole moments of the bonds. This has been quantified in an empirical bond polarizability model^28^ which can quantitatively predict the Raman intensities of fullerene^28^ and graphene ribbons^29^. In this approach, the polarizability is written as a sum of individual bond polarizabilities, which are assumed to be roughly independent of the chemical environment^29^:


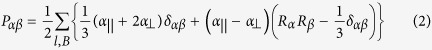


where 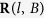 is the bond vector connecting atom *l* to one of its nearest neighbor atoms *l′* connected by bond B, the vector being normalized to unity. 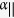 and 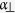 are the static longitudinal and perpendicular bond polarizability, respectively, which are further assumed to only depend on the bond length *R*. We note that equation (2) has further assumed cylindrical symmetry around the principal axis of each bond – although this may not be exactly correct in general systems, in this work, we are only concerned with relative magnitudes of the Raman intensities, for which these details become unimportant (see later discussion). The derivative of the bond polarizability 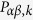, which determines the Raman intensity, can be written as:


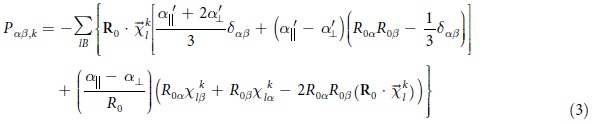


where 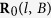 is the bond vector at equilibrium configuration, normalized to unity, *R*_0β_ is the β component of 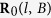, while *R*_0_ is the bond length at equilibrium. 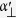 and 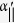 are the radial derivatives of the bond polarizability with respect to the bond length. For the 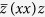 configuration, we have:


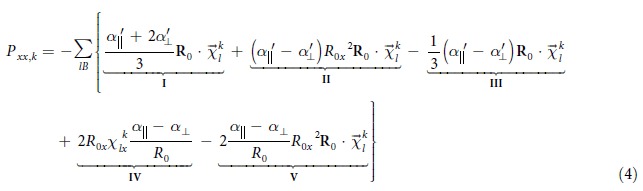


For the sake of clarity in explanation, we note that the RHS of equation (4) has five terms, which we will analyse in detail later.

**Figure 3:**
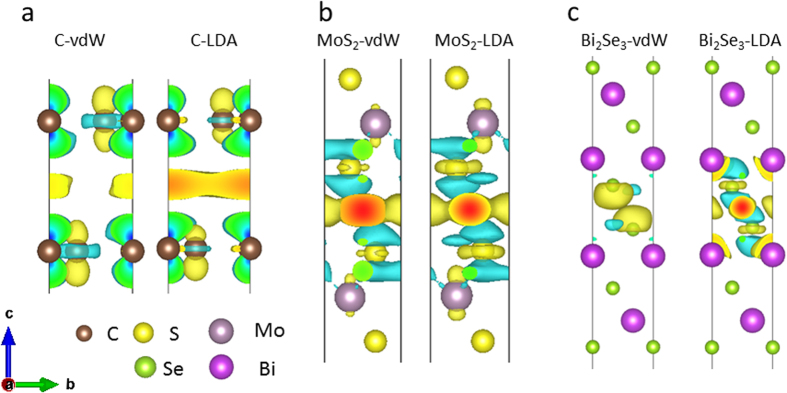
LDA and vdW calculated charge density difference for bilayer (a) graphene, (b) MoS_2_ and (c) Bi_2_Se_3_ at the equilibrium distance. The charge accumulation and depletion is denoted by the yellow and blue color, respectively.

Here, we apply the above bond polarizability model to the interlayer vibrations in layered materials. The justification for applying the bond polarizability model to the interlayer modes is shown in Fig. 3, in which we find distinct charge accumulation in the interlayer regions along the axes connecting nearest neighbouring atoms, for bilayer graphene, MoS_2_ and Bi_2_Se_3_. This indicates that although the interlayer interactions are widely believed to be of the van der Waals type, they also have some covalent character. The charge accumulated in these weak covalent bonds can produce dipole moments in the presence of an applied electric field imposed by incident radiation, resulting in Raman intensities which depend on the bond direction, i.e. the stacking order. It is because of this small amount of covalency that experimentally, different interlayer modes are observed for differently stacked materials – if there is no covalency at all, but rather completely delocalized interlayer charge distributions, the stacking order would not have such a significant impact on the Raman intensities.

Before applying the bond polarizability model in full detail, we note that we can understand quite simply the effect of stacking order on Raman intensities of the interlayer modes by using the concept of interlayer bond polarizabilities. We shall focus on the shear mode which is of interest here. For systems with in-plane isotropy, such as graphene, we can without loss of generality consider the shear modes with layers moving in the *x* direction. When two adjacent layers move against each other, we consider how the *x* component of the dipole moments along all nearest neighboring atoms would change. For AB and ABC stacked materials such as graphene, TMDs and Bi_2_Se_3_, each atom will have one bond B* with the largest *x* component, and this will determine the effective direction of the *x* component dipole moment induced by the field. In AB stacked materials, B* is pointing in opposite directions as we move from layer to layer. However, in ABC stacked materials, B* is pointing in the same direction moving from layer to layer. Therefore to maximize the change in dipole moment (for the largest Raman intensity), adjacent layers should be moving completely out-of-phase for AB stacked systems, while the whole system should be stretched out in the *x* direction (like a deck of cards) for ABC stacked systems. This suggests that S_N-1_ and S_1_ should have the largest Raman intensities in AB and ABC stacked systems respectively, consistent with the above discussions.

**Figure 4:**
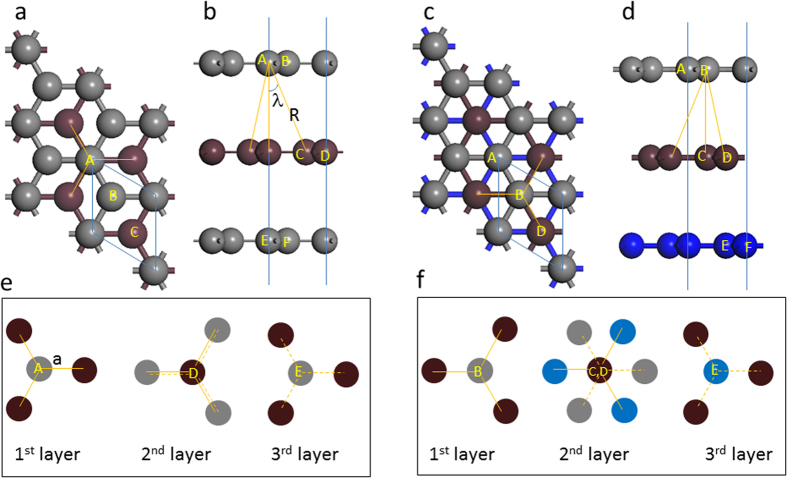
Top view and side view of the (a,b) AB stacked 3LG and (c,d) ABC stacked 3LG. The gray and brown balls respectively represent the C atoms in the odd and even numbered layers in AB stacked 3LG, while gray, brown and blue balls represent C atoms in the first, second and third layer of ABC stacked 3LG, respectively. (**e**) and (**f**) show the top view of the projected bonds for each layers. The solid and dash lines refer respectively to bonds connected downward and upward. The unit cell of 3LG is shown by the blue diamond.

**Figure 5:**
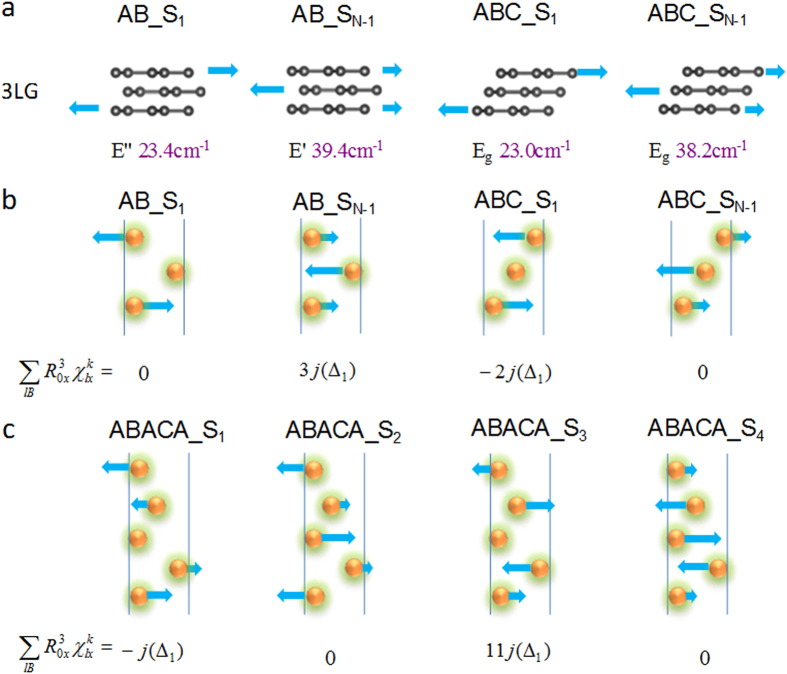
(**a**) Atomic displacements of the interlayer shear modes in AB and ABC stacked 3LG. (**b**) General bond polarizability model in AB and ABC stacked 3LG, a sphere represents a graphene layer. (**c**) The application of general bond polarizability model to ABACA stacked 5 layer MoSe_2_.

We now illustrate the above qualitative picture more rigorously using equation (4) for AB and ABC stacked three layer graphene (3LG) (Fig. 4). We shall sum over all interlayer bonds with bond length not larger than R for the atoms in the unit cell (the intralayer bonds will not contribute to the change in polarization since the atoms within each layer move in phase). The largest *x* component of the interlayer bond is *a* = R*sinλ (see Fig. 4). We note that 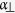, 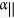, 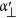 and 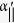 are all constants. Considering the interlayer shear mode with layers moving in the *x* direction, only the *x* component of 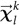 is non-zero. We can now see that terms I, III and IV are proportional to 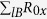 which is equal to zero from symmetry. On the other hand, terms II and V are proportional to 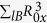 which is non-zero. For example, by summing the *x* component of the interlayer bonds connected to atom A in the AB stacked 3LG shown in Fig. 4a,b, we have 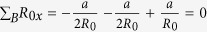, 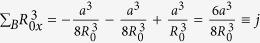. Since the B atom of the first layer is in the center of the hexagon of the second layer, both 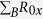 and 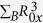 will be zero, giving zero contribution for all terms in equation (4). We plot in Fig. 4e–f the layer by layer top view of the interlayer bonds that will give non-zero contributions to equation (4). For AB stacked 3LG, the non-zero 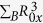 term is equal to *j*, −2*j* and *j* for atoms A, D and E respectively, while for ABC stacked 3LG, 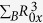 is equal to −*j*, 0 and *j* for atoms B, C/D and E respectively. Next to evaluate the non-zero terms II and V, we need to multiply 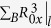 by 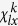 for each atom *l* in the unit cell and sum over *l*. As shown in Fig. 5a, the atomic displacements of the interlayer shear modes are the same for AB and ABC stacked 3LG. Setting 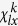 to be Δ_1_, Δ_2_ and Δ_3_ for atoms in the 1^st^, 2^nd^ and 3^rd^ layers respectively, we obtain:


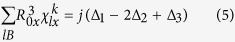


for ABstacked 3LG, and


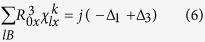


for ABC stacked 3LG.

For the S_1_ mode (lowest frequency) in 3LG, Δ_1_ = −Δ_3_, and Δ_2_ = 0. From equation (5), 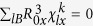 and therefore the Raman intensity is zero in AB stacked 3LG, while from equation (6), 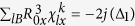 for ABC stacked 3LG. Thus, from this model, we can explain why, although the lowest frequency modes are Raman active in both AB and ABC stacked FLG, only the lowest frequency shear mode in ABC stacked FLG has non-zero Raman intensity. A similar analysis applies to the highest frequency S_N-1_ mode.

The Raman intensities estimated from the bond polarizability model compare well with LDA calculated intensities for the interlayer shear modes of 2–7 L graphene and bulk graphite (Supplementary Figure S2). In AB stacked bulk graphite, there is a Raman peak located around 44 cm^−1^ corresponding to the 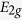 mode, but no Raman peaks are found in ABC stacked bulk graphite in the low frequency range. In the limit of ABC stacked bulk graphite, we find that the individual bond polarizabilities for the interlayer modes will always cancel out when the periodic boundary condition is considered, thus giving zero Raman intensity.

For the breathing modes, term IV in equation (4) is zero, but in general the other terms are non-zero. However, since differences in stacking order are reflected in differences in the in-plane components of 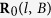, while it is the *z* component of 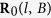 that shows up in equation (4), the stacking order has no influence on the relative Raman intensities of the breathing modes, consistent with our DFT calculations in the previous section.

### Generalization to other 2D materials

Besides graphene multilayer systems, similar stacking dependent interlayer vibration modes are expected to exist in other 2D layered materials, such as MoSe_2_, MoS_2_, WSe_2_ and Bi_2_Se_3_. The building block of TMD is made up of three atomic layers (trilayer), with the transition metal atom covalently bonded to the chalcogen atom within the trilayer, while the building block of Bi_2_Se_3_ consists of “quintuple layers”. In the interlayer vibration modes, the atoms within the building block are moving in phase with similar displacements, so the interlayer modes represent the relative displacements between the building blocks separated by the interlayer gap. Similar to graphite, the most stable structure for MoSe_2_, MoS_2_ and WSe_2_ is the 2H (AB) stacked sequence, with the rhombohedral (ABC) stacked system being a metastable structure. However, for Bi_2_Se_3_, the stable structure has the rhombohedral (ABC) stacked order. The symmetry of AB stacked TMD is the same as that for AB stacked FLG, while both ABC stacked multilayer Bi_2_Se_3_ and ABC stacked FLG belong to the symmorphic space group 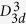 (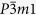) with inversion symmetry. In contrast, ABC stacked TMD belongs to the point group 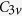 without inversion symmetry, with the irreducible representations of zone center phonon modes given by 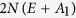.

**Figure 6:**
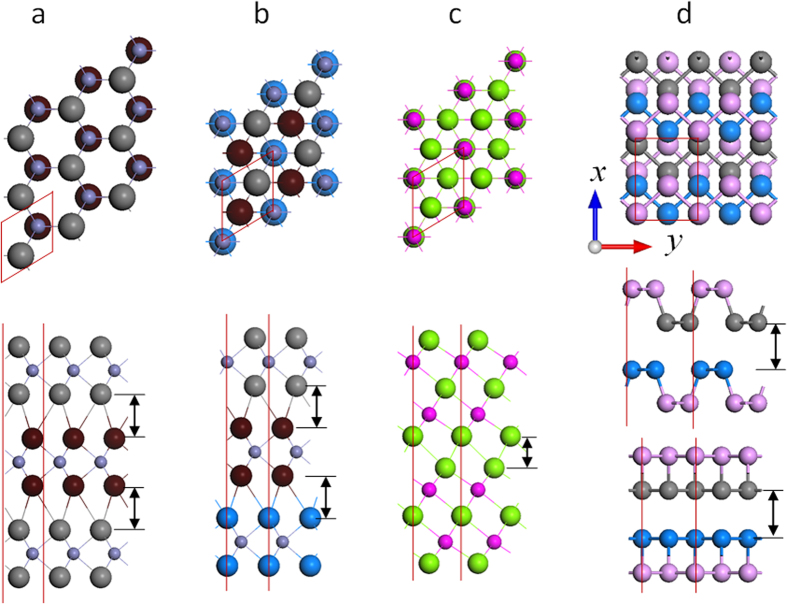
Top view and side view of (a) AB stacked (b) ABC stacked MoS_2_, (c) Bi_2_Se_3_ and (d) Black phosphorus. To have a better visual effect, the S atoms in odd and even numbered layers are shown in grey and brown in AB stacked MoS_2_, while in ABC stacked MoS_2_, they are shown in grey, brown, and blue for 1^st^, 2^nd^ and 3^rd^ layers respectively. The P atoms near the interlayer gap are plotted in grey and blue. The interlayer gaps are denoted by black double arrows.

**Figure 7:**
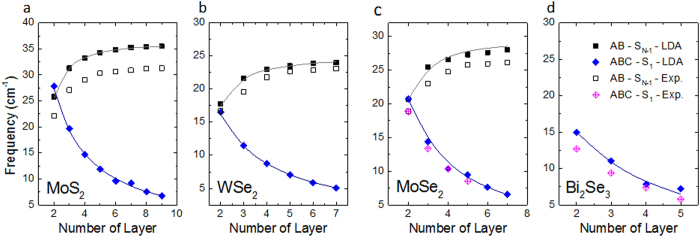
Frequency evolution trends of interlayer shear modes with largest Raman intensity in (a) MoS_2_, (b) WSe_2_, (c) MoSe_2_ and (d) Bi_2_Se_3_ with different stacking orders. The LDA calculated data are plotted with solid dots and the experimental data are shown in empty dots. The gray and indigo lines result from fitting of the largest frequency shear modes (S_N-1_) and the lowest frequency shear modes (S_1_) for AB and ABC stacking order, respectively, using the linear chain model^12^. The resulting force constants are shown in Table S10.

We note that in both TMDs and Bi_2_Se_3_, the interlayer bonds are the same as those represented by the A atom in Fig. 4 (except for the vertical bond between A and the atom below it). For example, each chalcogen atom has three nearest neighbouring chalcogen atoms in the adjacent layer, and from the top view, it sits in the middle of the triangle formed by these three neighbours (Fig. 6a,b). Therefore the above bond polarizability analysis for FLG also applies to these systems. Indeed, according to our DFT LDA calculations for AB and ABC stacked TMDs as well as for Bi_2_Se_3_ (ABC stacking), the shear modes with largest Raman intensity are S_N-1_ and S_1_ for AB and ABC stacked systems respectively (Fig. 7). Detailed LDA results are shown in Supplementary Tables S3 to S10.

### Experimental evidence

**Figure 8:**
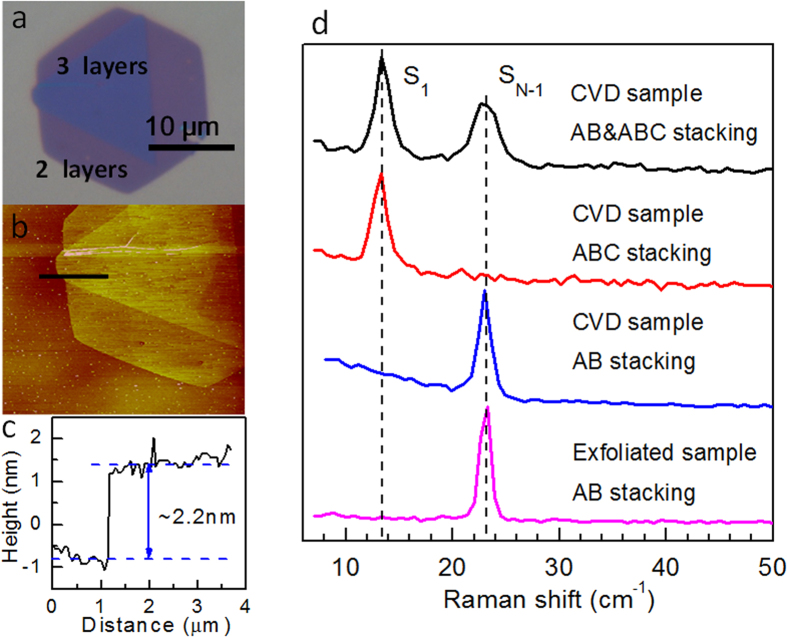
(**a**) Optical and (**b**) AFM image of a 3L MoSe_2_ sample, the cross cection height is shown in (**c**). (**d**) Raman spectra of the 3L MoSe_2_ with different stacking orders.

Next, we turn to experimental evidence of the predicted trends. Recently, the group of Rui He has reported the Raman spectra of suspended AB and ABC stacked trilayer graphene (3LG)^30^. At the ultralow frequency range, they observed an interlayer breathing mode (59 cm^−1^) and highest frequency shear mode (37 cm^−1^) in AB stacked three-layer graphene (3LG), and the same breathing mode (59 cm^−1^) and the lowest frequency shear mode (22 cm^−1^) in ABC stacked 3LG. This observation is in good agreement with our analysis for the 3LG system. Our experiments on 5LG also found that the highest frequency shear mode (42 cm^−1^) only appears in AB stacked 5LG but disappears in ABC stacked 5LG (see Supplementary Figure S3). For the other 2D materials, besides the experimental results already published for AB stacked MoS_2_ and WSe_2_, and for ABC stacked Bi_2_Se_3_ (shown in Fig. 7), we have measured the ultralow Raman spectra for AB and ABC stacked 3L MoSe_2_ (Fig. 8).

In Fig. 8, we show results from Raman scattering experiments on AB and ABC stacked MoSe_2_ samples grown from chemical vapor deposition (CVD)^31^. Figure 8a–c shows the optical contrast and atomic force microscopy (AFM) image of a 3L MoSe_2_ sample; the measured Raman spectra on different 3L MoSe_2_ samples are shown in Fig. 8d. Interestingly, 35% of all 43 samples exhibit only a peak around 13.3 cm^−1^, very close to the theoretical prediction of 14.4 cm^−1^ for the lowest frequency shear mode S_1_ in 3L ABC stacked MoSe_2_ samples, while 28% of all samples exhibit a peak at 23.1 cm^−1^, close to our theoretically predicted value of 25.5 cm^−1^ for the highest frequency shear mode S_N-1_ in 3L AB stacked MoSe_2_. Since exfoliated 2H MoSe_2_ has the AB stacking order, we compare our results with Raman spectra on the exfoliated sample. The Raman peak of the exfoliated AB stacked sample is around 23.3 cm^−1^, confirming that the samples that show a peak around 23.1 cm^−1^ are AB stacked. It is noted that 37% of all samples detect both S_1_ and S_N-1_ modes, because the spot size of our laser is at least 1μm, and the 2H and 3R phases can coexist in the same sample with either a sharp boundary^32^, or a few-hundred nanometer wide transition area^31^. Furthermore, scanning tunneling electron microscopy (STEM) experiments performed on the CVD-grown samples^31^,^32^ can be used to distinguish the AB and ABC 3L stacked samples by comparing the STEM images with simulated images^31^. Experimental data for MoSe_2_ thin films with different number of layers is plotted in Fig. 7c; the results agree well with the theoretical predictions.

## Discussion

The bond polarizability model is based on the formula for nonresonant Raman scattering intensities, which are typically computed within first principles density functional perturbation theory within the Placzek approximation. The computed *non-resonant* Raman intensities match reasonably well with intensities in experimental ultralow frequency Raman spectra obtained for ABA and ABC stacked materials with typical laser wavelengths of 532 nm^12^,^14^,^30^,^31^,^33^. Recently, resonant Raman scattering of interlayer vibration mode has been observed in the twisted few layer graphene system^34^,^35^,^36^,^37^. It is noted that the symmetry of the system is much lowered because of the twist, so that many more modes will become Raman active. On the other hand, the twist between two graphene layers results in an overlap of the two Dirac cones in a small region of k-space, leading to Van Hove singularities in the joint density of states, which allow for more optically allowed transitions^35^,^38^,^39^. Due to electron-phonon coupling, Raman intensities are enhanced when the laser excitation wavelength matches those of the optical transitions^38^. While resonant Raman scattering reveals new insights into 2D layered materials^16^, our analysis and conclusions are still applicable in the majority of ultralow frequency non-resonant Raman experiments, and in particular for simply AB and ABC stacked layered materials^12^,^13^,^14^,^21^,^23^,^24^,^30^,^31^,^33^,^40^,^41^,^42^, as we have discussed above.

The advantage of a bond polarizability model is that it can be efficiently applied to more complicated stacking sequences in thicker samples, such as the ABACA stacking in 5 layer MoSe_2_. We show these predictions in Fig. 5c. As the ABACA stacking order could not be distinguished in STEM, the predicted Raman intensities along with experimental Raman spectra were used to assign the stacking sequence in a few of our CVD-grown 5 layer MoSe_2_ samples^31^. We also note that the bond polarizability predictions are consistent with our LDA results for these complicated stacking sequences. The above results provide evidence that although the space groups are not all the same for materials with the same stacking order, the frequency evolution trends as predicted by our bond polarizability model are general. Furthermore, these predictions can be used to determine the stacking orders in experimental samples, even when the stacking order cannot be distinguished by microscopy.

Finally, we note that in few layer black phosphorus (Fig. 6d), the in-plane shear modes cannot be detected under the 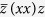 polarization configuration. Considering both shear modes in the *x* and *y* directions, we can see that any change in bond polarizability will cancel out when we sum over all nearest neighbor bonds, because of the mirror planes along the *y* and *x* directions respectively. On the other hand, the breathing modes can have non-zero Raman intensity in the 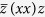 configuration. These predictions are consistent with first principles calculations and Raman spectroscopy experiments^40^.

In conclusion, we have shown that the interlayer bond polarizabilities can allow us to understand, both intuitively and semi-quantitatively, the Raman intensities of interlayer modes in general 2D layered materials. Specifically we find that the change in polarizability is maximized for the lowest frequency shear mode in ABC stacked materials, but for the highest frequency shear mode in AB stacked materials, regardless of the details of the space group. The resultant differences in Raman intensity result in clear and distinct frequency trends for AB and ABC stacked systems – as the number of layers increases, the interlayer shear mode red shifts for ABC stacked systems, and blue shifts for AB stacked systems. Because these trends are distinct, and furthermore, do not overlap, they provide a general way to distinguish AB and ABC stacking in 2D layered materials. This bond polarizability model also provides consistent results when applied to interlayer modes in other materials such as black phosphorus, and can be used as a tool to make quick predictions on Raman intensities for more complicated stacking orders. Our predictions are substantiated with extensive first principles calculations as well as Raman spectroscopy measurements on different 2D materials.

## Methods

First principles calculations of vibrational Raman spectra are performed within density-functional perturbation theory (DFPT) as implemented in the plane-wave code QUANTUM-ESPRESSO^43^. The local density approximation (LDA)^44^ to the exchange-correlation functional with projector-augmented wave potentials is employed for the calculation of phonon frequencies, while the non-resonant Raman intensity is calculated using the norm-conserving pseudopotential, within the Placzek approximation^26^. With the frequency and Raman intensity, a scale parameter of 1 is used in the Lorentzian broadening function to get the calculated Raman spectrum. To get the converged results, a plane-wave kinetic energy cutoff of 65 Ry is used for the wave functions, and the convergence threshold is set to 10^−9 ^eV and 10^−18 ^eV in the electron and phonon self-consistent calculation, respectively. The structures are considered as relaxed when the maximum component of the Hellmann-Feynman force acting on each atom is less than 0.003 eV/Å. A Monkhorst-Pack k-point mesh of 44 × 44 × 1, 17 × 17 × 1 and 11 × 11 × 1 are used to sample the Brillouin Zones for the FLG, TMD and Bi_2_Se_3_ systems, respectively. We use a vacuum thickness of 16 Å in the direction perpendicular to the slabs to prevent interactions between periodic slab images. The spin-orbit coupling effect is included self-consistently by using fully relativistic pseudopotentials for the valence electrons in Bi_2_Se_3_.

Experimentally, the graphene layers and MoSe_2_ are prepared by the mechanical exfoliation method^45^ and chemical vapor deposition method^32^, respectively. The Raman spectroscopy measurements are conducted in a backscattering geometry, excited with a Helium-Neon laser with λ = 532 nm for MoSe_2_ and graphene layers. The detection of ultralow frequency is achieved by filtering out the laser side bands through the adoption of a reflecting Bragg grating, the ultralow frequency of MoSe_2_ is achieved by triple-grating setup (Horiba-JY T64000). The laser power is kept below 0.05mW on the sample surface to avoid laser-induced heating.

## Additional Information

**How to cite this article**: Luo, X. *et al.* Stacking sequence determines Raman intensities of observed interlayer shear modes in 2D layered materials – A general bond polarizability model. *Sci. Rep.* **5**, 14565; doi: 10.1038/srep14565 (2015).

## Supplementary Material

Supplementary Information

## Figures and Tables

**Table 1:** The space group of the multilayer graphene, MoS_2_ and Bi_2_Se_3_ under AB and ABC staking order.

Number of layers	Singlelayer	AB stacking		ABC stacking	
		OddNumber ofLayers	EvenNumber ofLayers	OddNumber ofLayers	EvenNumber ofLayers
Multi-layered Graphene	D^1^_6h_	D^1^_3h_	D^3^_3d_	D^3^_3d_	D^3^_3d_
MoS_2_ (other 2H TMD)	D^1^_3h_	D^1^_3h_	D^3^_3d_	C^1^_3v_	C^1^_3v_
Bi_2_Se_3_ (Bi_2_Te_3_)	D^3^_3d_	−	−	D^3^_3d_	D^3^_3d_
