# Supplementary material for: Stacking sequence determines Raman intensities of observed interlayer shear modes in 2D layered materials – A general bond polarizability model
Source: Sci Rep. 2015 Oct 15;5:14565. doi: 10.1038/srep14565 (PMC4606731; doi:10.1038/srep14565)
Supplement: Supplementary Information [file srep14565-s1.pdf]

Supporting Information for

Stacking sequence determines Raman intensities of observed interlayer shear modes in 2D layered materials – A general bond polarizability model

Xin Luo, [⊥,&,£] *Xin Lu,* [†] *Chunxiao Cong,* [†] *Ting Yu,* [†,⊥] *Qihua Xiong* [†,₸] *and Su Ying Quek* [⊥,&,£,*]

[⊥]Centre for Advanced 2D Materials and Graphene Research Centre
National University of Singapore, 6 Science Drive 2, Singapore 117546

[£]Department of Physics, National University of Singapore, 2 Science Drive 3, Singapore 117551

[&]Institute of High Performance Computing, 1 Fusionopolis Way, #16-16 Connexis, Singapore 138632

[†]Division of Physics and Applied Physics, School of Physical and Mathematical Sciences, Nanyang Technological University, Singapore 637371

[₸]NOVITAS, Nanoelectronics Centre of Excellence, School of Electrical and Electronic Engineering, Nanyang Technological University, Singapore 639798

*To whom correspondence should be addressed. Email: phyqsy@nus.edu.sg

Contents

1) Figures

2) Raman measurement of ABA and ABC stacked five layer graphene

3) Group Theory Analysis

4) Tables

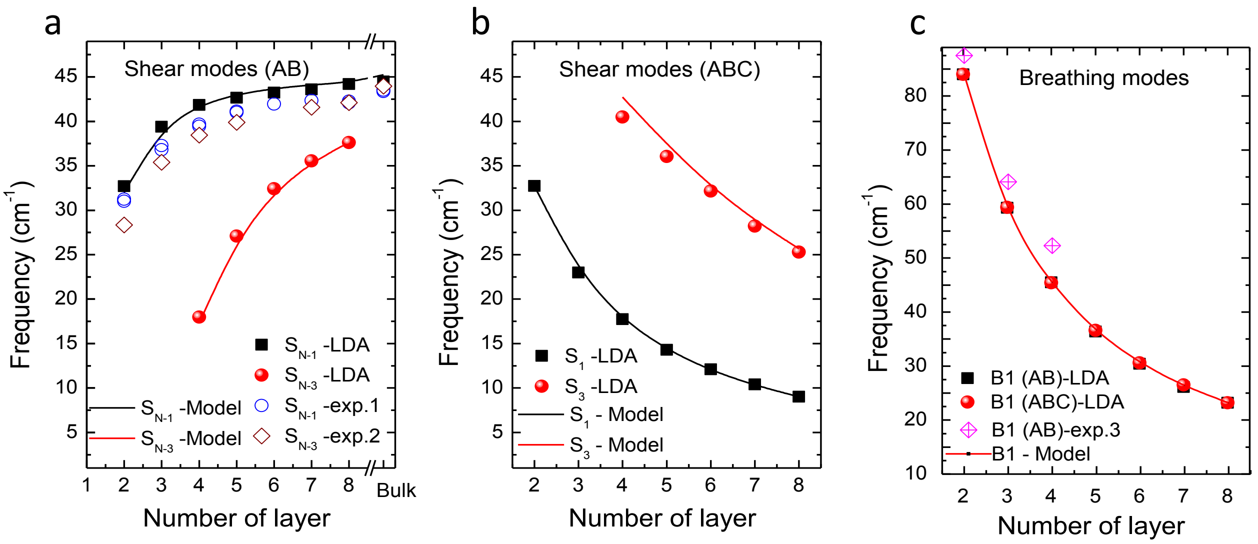

Figure S1. The calculated frequencies of Raman active shear modes in (a) AB and (b) ABC stacked FLG, while the breathing modes for both stacking orders are shown in (c). Experiments 1, 2, 3 are from Ref [1, 2, 3], respectively. The "Model" calculation here refers to a nearest neighbor linear chain model.[1] From this model, we find that the interlayer shear force constants k_x=1.40×10^{19} N/m^3 for AB stacked FLG and 1.46×10^{19} N/m^3 for ABC stacked FLG. The fact that these force constants are almost identical indicates that the stacking order does not have significant influence on the interlayer interaction strength. The interlayer breathing force constants k_z=9.63×10^{19} N/m^3 in both systems.

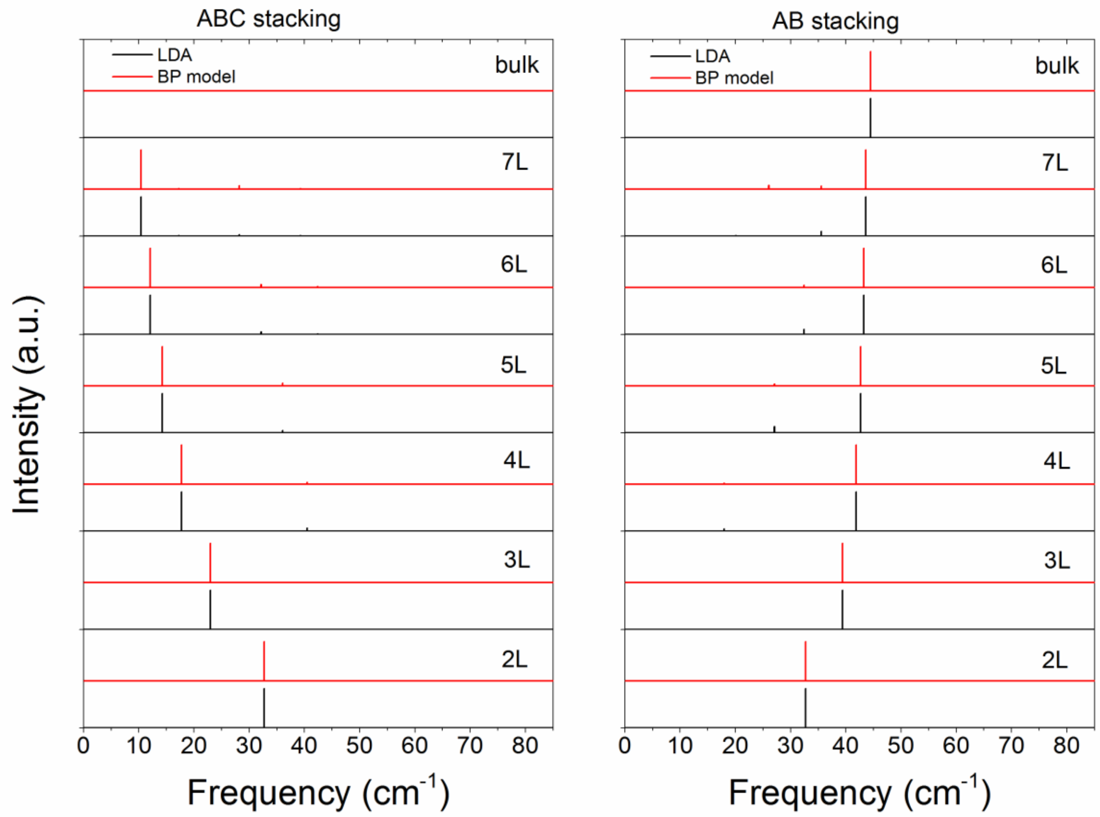

Figure S2. Comparison of the thicknees dependent Raman intensities of interlayer shear modes in the LDA calculation and bond polarizability (BP) model for ABC and AB stacked FLG.

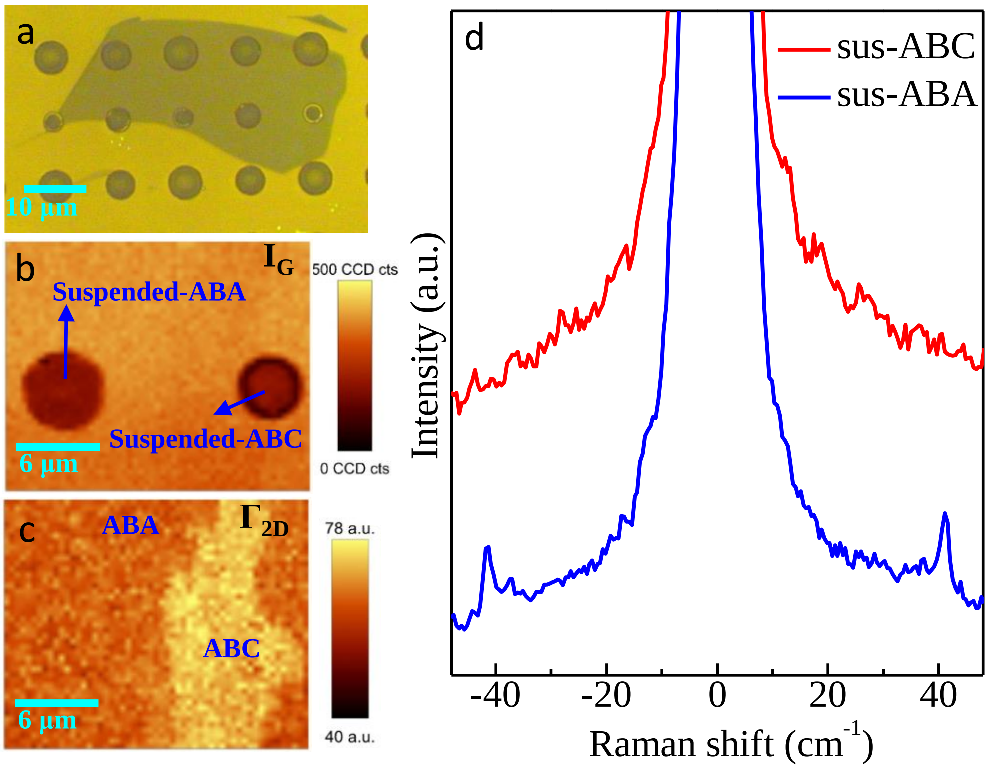

Figure S3. (a) Optical images of a 5 layer graphene (5LG) sample and the corresponding Raman images of (b) the G mode intensity and (c) the 2D mode linewidth. (d) Experimentally measured Raman spectra of 5LG.

Raman measurement of ABA and ABC stacked five layer graphene

Figure S3 shows the experimental results of a mechanically exfoliated 5LG on holed 300 nm SiO_2/Si substrates. The optical contrast image in Figure S3a shows that the sample is of the same thickness as 5 layer graphene. We characterized the stacking order in AB and ABC stacked FLG by comparing the Raman images of the G mode intensity and the 2D mode width[4, 5]; it is clear that there are two different domains with different stacking order in the same sample. Since the spectral band width is much larger in the ABC stacked compared with the AB stacked order in our experimental setting, we assign the much brighter part to ABC stacking, while the darker to AB stacking. The low frequency Raman spectra are then measured for the suspended sample above the hole. For the AB stacked region, the Stokes and anti-Stokes Raman spectra in Figure S3d show a typical spectral feature peak of 5LG, i.e., a shear mode is observed at 41.5 cm^{-1}. The Raman peak is in agreement with the reported value in other experiments[1], and in good agreement with our LDA predictions (43.2 cm^{-1}, corresponding to S_{N-1}). In contrast, our Raman spectroscopy did not detect any noticeable peak near 41 cm^{-1} in the ABC stacked region, in agreement with our bond polarizability analysis and the LDA calculation. The calculation predicts the S_1 peak should be detected near 14 cm^{-1} for the ABC stacked 5LG, however, the large background noise has merged the feature peaks of the 5LG below 15 cm^{-1}. Thus this experimental result has partially corroborated the LDA calculations, and is consistent with the general bond polarizability predictions.

Group theory analysis

Here we analyze the phonon modes and their Raman tensors of AB and ABC stacked few-layer graphene (FLG) from group theory. A vibrational phonon mode that can be detected by Raman spectroscopy must satisfy the fundamental Raman selection rule. It is noted that some overtones that are forbidden in Raman spectra under non-resonant scattering will become weakly allowed when the resonant scattering effect is taking into account.[6]

The SLG and the most common AB stacked graphite belong to point group D_{6h}, so they share the same irreducible representation for the phonon normal modes at Γ: $A_{2u}+E_{1u}+E_{2g}+B_{2g}$, among which E_{2g} is Raman active, A_{2u} and E_{1u} are IR active, and B_{2g} is optically inactive. AB stacked N-layer FLG belongs to point group D_{3h} (space group D_{3h}^{1}) for N odd. ABC stacked FLG (including ABC stacked graphite) and AB stacked N-layer FLG (N even) belong to the point group D_{3d} (space group D_{3d}^{3}) with inversion symmetry. The mutual exclusion applies to the structures with inversion symmetry: a vibrational mode may be either IR active or Raman active but not both. As a result, the irreducible representations of zone center phonon modes in odd number AB stacked FLG are: $(N-1)(A_1'+A_2''+E'+E'')+2A_2''+2E'$, and those in even number AB stacked FLG and all the ABC stacked FLG are: $N(A_{1g}+A_{2u}+E_g+E_u)$. The A_1', A_{1g}, E'' and E_g modes are Raman active, A_2'', A_{2u} and E_u modes are IR active, E' is both Raman and IR active. Here, the two fold degenerate *E* symmetry mode represents the in-plane vibration mode, and the non-degenerate *A* symmetry mode represents the out-of plane vibration mode along the *c* axis. From group theory, the Raman tensors of above Raman active modes have quadratic forms:

$$A_1'=A_{1g}=\begin{pmatrix} a & 0 & 0 \\ 0 & a & 0 \\ 0 & 0 & c \end{pmatrix}, E''=\begin{pmatrix} 0 & 0 & a \\ 0 & 0 & b \\ a & b & 0 \end{pmatrix}, E'=E_{2g}=\begin{pmatrix} a & c & 0 \\ c & -a & 0 \\ 0 & 0 & 0 \end{pmatrix}, E_g=\begin{pmatrix} a & c & d \\ c & -a & f \\ d & f & 0 \end{pmatrix}. \quad (1)$$

All the irreducible representation and optical activity of the interlayer vibration modes in 2-8LG are listed in Tables S-1 and S2.

Table S1. LDA calculated Γ point phonon frequencies (cm^{-1}) and relative Raman intensities *Ixx* (shown in parentheses) of the interlayer vibration modes in AB stacked 2-8LG and bulk graphite. The intensity is normalized by the largest value in each thickness. The irreducible representation and Raman [R]/infrared [IR] activity are also indicated; IR+R indicate the mode is both Raman and infrared active, while Ina indicates that the mode is optically inactive. The background colors indicate the largest intensity shear and breathing modes, respectively.

LDA calculation	AB stacking few layer graphene							
Thickness	bulk	8	7	6	5	4	3	2
	44.49 (1.0)<br>E_{2g} [R]	9.1 (3e-4)<br>E_g [R]	10.3 (0)<br>E'' [R]	12.0 (0.002)<br>E_g [R]	14.3 (0)<br>E'' [R]	18.0 (0.06)<br>E_g [R]	23.4 (0)<br>E'' [R]	32.7 (1.0)<br>E_g [R]
		17.6 (0)<br>E_u [IR]	20.1 (0.02)<br>E' [IR+R]	23.0 (0)<br>E_u [IR]	27.1 (0.17)<br>E' [IR+R]	32.6 (0)<br>E_u [IR]	39.4 (1.0)<br>E' [IR+R]	
		25.6 (0.04)<br>E_g [R]	28.6 (0)<br>E'' [R]	32.4 (0.13)<br>E_g [R]	36.8 (0)<br>E'' [R]	41.8 (1.0)<br>E_g [R]		
shear modes		32.4 (0)<br>E_u [IR]	35.5 (0.13)<br>E' [IR+R]	39.1 (0)<br>E_u [IR]	42.7 (1.0)<br>E' [IR+R]			
		37.6 (0.16)<br>E_g [R]	40.6 (0)<br>E'' [R]	43.2 (1.0)<br>E_g [R]				
		41.4 (0)<br>E_u [IR]	43.6 (1.0)<br>E' [IR+R]					
		44.2 (1.0)<br>E_g [R]						
	115.82 (0)<br>B_{2g} [Ina]	23.2 (0.009)<br>A_{1g} [R]	26.1 (0.009)<br>A'1 [R]	30.4 (0.008)<br>A_{1g} [R]	36.4 (0.008)<br>A'_1 [R]	45.5 (0.007)<br>A_{1g} [R]	59.3 (0.007)<br>A'_1 [R]	84.0 (0.01)<br>A_{1g} [R]
		44.7 (0)<br>A_{2u} [IR]	51.0 (0)<br>A''_2 [IR]	58.8 (0)<br>A_{2u} [IR]	69.2 (0)<br>A''_2 [IR]	83.7 (0)<br>A_{2u} [IR]	102.3 (0)<br>A''_2 [IR]	
		65.3 (6e-4)<br>A_{1g} [R]	73.2 (7e-4)<br>A'_1 [R]	83.0 (9e-4)<br>A_{1g} [R]	95.0 (0.002)<br>A'_1 [R]	109.0 (0.005)<br>A_{1g} [R]		
breathing modes		83.2 (0)<br>A_{2u} [IR]	91.6 (0)<br>A''_2 [IR]	101.3 (0)<br>A_{2u} [IR]	111.0 (0)<br>A''_2 [IR]			
		97.1 (0.001)<br>A_{1g} [R]	105.2 (0.002)<br>A'_1 [R]	112.5 (0.002)<br>A_{1g} [R]				
		107.2 (0)<br>A_{2u} [IR]	113.5 (0)<br>A''_2 [IR]					
		115.6 (6e-4)<br>A_{1g} [R]						

Table S2. LDA calculated Γ point phonon frequencies (cm^{-1}) and relative Raman intensities I_{xx} (shown in parentheses) of the interlayer vibration modes in ABC stacked 2-8LG and bulk graphite. The notations follow Table S1.

LDA calculation	ABC stacked few layer graphene							
Thickness	bulk	8	7	6	5	4	3	2
shear modes	45.04 (7e-9) E_g [R]	9.0 (1.0) E_g [R]	10.4 (1.0) E_g [R]	12.1 (1.0) E_g [R]	14.3 (1.0) E_g [R]	17.8 (1.0) E_g [R]	23.0 (1.0) E_g [R]	32.7 (1.0) E_g [R]
	45.05 (0) E_u [IR]	13.5 (0) E_u [IR]	17.3 (0) E_u [IR]	21.1 (0) E_u [IR]	25.6 (0) E_u [IR]	31.3 (0) E_u [IR]	38.2 (0) E_u [IR]	
		25.3 (0.09) E_g [R]	28.2 (0.05) E_g [R]	32.2 (0.07) E_g [R]	36.0 (0.06) E_g [R]	40.5 (0.09) E_g [R]		
		28.9 (0) E_u [IR]	33.3 (0) E_u [IR]	37.6 (0) E_u [IR]	41.2 (0) E_u [IR]			
		36.8 (0.02) E_g [R]	39.3 (0.02) E_g [R]	42.4 (0.02) E_g [R]				
		39.6 (0) E_u [IR]	42.3 (0) E_u [IR]					
		42.5 (0.006) E_g [R]						
breathing modes	127.99 (6e-9) A_{1g} [R]	23.2 (0.03) A_{1g} [R]	26.5 (0.02) A_{1g} [R]	30.6 (0.03) A_{1g} [R]	36.6 (0.02) A_{1g} [R]	45.4 (0.02) A_{1g} [R]	59.4 (0.01) A_{1g} [R]	84.0 (0.01) A_{1g} [R]
	128.0 (0) A_{2u} [IR]	45.4 (0) A_{2u} [IR]	50.9 (0) A_{2u} [IR]	59.1 (0) A_{2u} [IR]	69.4 (0) A_{2u} [IR]	83.3 (0) A_{2u} [IR]	101.6 (0) A_{2u} [IR]	
		65.6 (9e-4) A_{1g} [R]	73.3 (4e-4) A_{1g} [R]	83.2 (5e-4) A_{1g} [R]	94.9 (1e-4) A_{1g} [R]	107.6 (6e-6) A_{1g} [R]		
		83.2 (0) A_{2u} [IR]	91.4 (0) A_{2u} [IR]	101.2 (0) A_{2u} [IR]	110.6 (0) A_{2u} [IR]			
		97.4 (8e-6) A_{1g} [R]	104.3 (4e-7) A_{1g} [R]	112.4 (2e-5) A_{1g} [R]				
		107.95 (0) A_{2u} [IR]	114.3 (0) A_{2u} [IR]					
		114.6 (8e-6) A_{1g} [R]						

Table S3. LDA calculated Γ point phonon frequencies (cm^{-1}) and relative Raman intensities I_{xx} (shown in parentheses) of the interlayer vibration modes in AB stacked 2-9 TL and bulk MoS_2. AB stacked MoS_2 belongs to the same point group as AB stacked FLG, so their notations are the same as in Table S1.

LDA calculation	AB stacked few trilayer MoS_2								
Thickness	bulk	9	8	7	6	5	4	3	2
shear modes	35.7 (1.0)<br>E_{2g} [R]	6.0 (0)<br>E'' [R]	7.2 (0.02)<br>E_g [R]	7.9 (0)<br>E'' [R]	9.3 (0.006)<br>E_g [R]	11.0 (0)<br>E'' [R]	13.7 (0.02)<br>E_g [R]	18.3 (0)<br>E'' [R]	25.8 (0.03)<br>E_g [R]
		12.2 (0.008)<br>E' [IR+R]	13.7 (0)<br>E_u [IR]	15.5 (0.006)<br>E' [IR+R]	18.0 (0)<br>E_u [IR]	21.2 (5e-4)<br>E' [IR+R]	25.4 (0)<br>E_u [IR]	31.3 (0.08)<br>E' [IR+R]	
		17.8 (0)<br>E'' [R]	20.0 (1e-4)<br>E_g [R]	22.3 (0)<br>E'' [R]	25.4 (0.002)<br>E_g [R]	29.2 (0)<br>E'' [R]	33.3(0.13)<br>E_g [R]		
		23.1 (3e-5)<br>E' [IR+R]	25.4 (0)<br>E_u [IR]	28.0 (0.004)<br>E' [IR+R]	31.2 (0)<br>E_u [IR]	34.3 (0.12)<br>E' [IR+R]			
		27.5 (0)<br>E'' [R]	30.0 (0.006)<br>E_g [R]	32.4 (0)<br>E'' [R]	34.8 (0.15)<br>E_g [R]				
		31.2 (0.008)<br>E' [IR+R]	33.3 (0)<br>E_u [IR]	35.3 (0.14)<br>E' [IR+R]					
		33.9 (0)<br>E'' [R]	35.4 (0.08)<br>E_g [R]						
		35.5 (0.14)<br>E' [IR+R]							
breathing modes	57.9 (0)<br>B_{2g} [Ina]	9.6 (1.0)<br>A'1 [R]	10.7 (1.0)<br>A_{1g} [R]	12.9 (1.0)<br>A'1 [R]	14.5 (1.0)<br>A_{1g} [R]	17.1 (1.0)<br>A'_1 [R]	22.3 (1.0)<br>A_{1g} [R]	28.8 (1.0)<br>A'_1 [R]	41.4 (1.0)<br>A_{1g} [R]
		20.0 (0)<br>A''_2 [IR]	22.3 (0)<br>A_{2u} [IR]	25.2 (0)<br>A''_2 [IR]	29.5 (0)<br>A_{2u} [IR]	34.1 (0)<br>A''_2 [IR]	41.5 (0)<br>A_{2u} [IR]	51.5 (0)<br>A''_2 [IR]	
		29.3 (0.09)<br>A'1 [R]	32.4 (0.08)<br>A_{1g} [R]	36.4 (0.08)<br>A'_1 [R]	41.6 (0.08)<br>A_{1g} [R]	47.5 (0.06)<br>A'_1 [R]	54.0 (0.03)<br>A_{1g} [R]		
		37.6 (0)<br>A''_2 [IR]	41.0 (0)<br>A_{2u} [IR]	45.5 (0)<br>A''_2 [IR]	50.8 (0)<br>A_{2u} [IR]	55.5 (0)<br>A''_2 [IR]			
		45.1 (0.03)<br>A'1 [R]	48.7 (0.02)<br>A_{1g} [R]	52.6 (0.01)<br>A'_1 [R]	56.6 (0.006)<br>A_{1g} [R]				
		50.8 (0)<br>A''_2 [IR]	54.2 (0)<br>A_{2u} [IR]	57.4 (0)<br>A''_2 [IR]					
		55.0 (0.006)<br>A'1 [R]	57.3 (0.002)<br>A_{1g} [R]						
		57.5 (0)<br>A''_2 [IR]							

Table S4. LDA calculated Γ point phonon frequencies (cm^{-1}) and relative Raman intensities I_{xx} (shown in parentheses) of the interlayer vibration modes in ABC stacked 2-9 TL and bulk MoS_2. ABC stacked MoS_2 belongs to point group C_{3V} without inversion symmetry, their shear and breathing modes are denoted by E and A_1, respectively, both are IR and Raman active.

LDA calculation	ABC stacked few trilayer MoS_2								
Thickness	bulk	9	8	7	6	5	4	3	2
shear modes	34.0 (1e-9) E [IR+R]	6.8 (0.17) E [IR+R]	7.5 (0.19) E [IR+R]	9.2 (0.19) E [IR+R]	9.6 (0.19) E [IR+R]	11.9 (0.19) E [IR+R]	14.7 (0.19) E [IR+R]	19.7 (0.19) E [IR+R]	27.8 (0.18) E [IR+R]
	34.0 (4e-9) E [IR+R]	13.3 (1e-7) E [IR+R]	14.8 (1e-6) E [IR+R]	17.0 (2e-7) E [IR+R]	19.4 (1e-7) E [IR+R]	22.8 (1e-7) E [IR+R]	27.6 (2e-6) E [IR+R]	34.1 (7e-6) E [IR+R]	
		19.5 (0.02) E [IR+R]	21.6 (0.02) E [IR+R]	24.5 (0.02) E [IR+R]	27.4 (0.02) E [IR+R]	31.6 (0.01) E [IR+R]	36.4 (0.007) E [IR+R]		
		25.0 (1e-6) E [IR+R]	27.5 (1e-6) E [IR+R]	30.7 (1e-6) E [IR+R]	33.9 (2e-6) E [IR+R]	37.2 (1e-6) E [IR+R]			
		29.8 (0.003) E [IR+R]	32.5 (0.004) E [IR+R]	35.4 (0.003) E [IR+R]	37.9 (0.001) E [IR+R]				
		33.9 (1e-6) E [IR+R]	36.3 (1e-6) E [IR+R]	38.0 (1e-6) E [IR+R]					
		36.9 (1e-4) E [IR+R]	38.4 (2e-4) E [IR+R]						
		38.6 (2e-7) E [IR+R]							
breathing modes	48.7 (1e-9) A_1 [IR+R]	10.1 (1.0) A_1 [IR+R]	10.7 (1.0) A_1 [IR+R]	12.3 (1.0) A_1 [IR+R]	14.1 (1.0) A_1 [IR+R]	17.2 (1.0) A_1 [IR+R]	21.5 (1.0) A_1 [IR+R]	28.8 (1.0) A_1 [IR+R]	40.1 (1.0) A_1 [IR+R]
	48.7 (2e-9) A_1 [IR+R]	19.2 (1e-4) A_1 [IR+R]	21.4 (1e-8) A_1 [IR+R]	24.1 (1e-9) A_1 [IR+R]	28.2 (1e-8) A_1 [IR+R]	33.0 (1e-9) A_1 [IR+R]	39.6 (1e-7) A_1 [IR+R]	49.1 (1e-7) A_1 [IR+R]	
		28.1 (0.09) A_1 [IR+R]	31.1 (0.09) A_1 [IR+R]	35.2 (0.09) A_1 [IR+R]	39.5 (0.08) A_1 [IR+R]	45.3 (0.07) A_1 [IR+R]	52.1 (0.04) A_1 [IR+R]		
		35.9 (1e-6) A_1 [IR+R]	39.6 (3e-8) A_1 [IR+R]	44.1 (1e-7) A_1 [IR+R]	48.9 (1e-7) A_1 [IR+R]	53.3 (1e-7) A_1 [IR+R]			
		42.9 (0.02) A_1 [IR+R]	46.7(0.02) A_1 [IR+R]	50.6 (0.01) A_1 [IR+R]	54.4 (0.006) A_1 [IR+R]				
		48.7 (2e-7) A_1 [IR+R]	52.1 (1e-7) A_1 [IR+R]	54.4 (1e-7) A_1 [IR+R]					
		52.9 (0.003) A_1 [IR+R]	55.1 (0.001) A_1 [IR+R]						
		55.2 (1e-5) A_1 [IR+R]							

Table S5 LDA calculated Γ point phonon frequencies (cm^{-1}) and relative Raman intensities I_{xx} (shown in parentheses) of the interlayer vibration modes in AB stacked 2-7TL and bulk $MoSe_2$. The notations follow Table S1.

LDA calculation	AB stacked few trilayer $MoSe_2$						
Thickness	bulk	7	6	5	4	3	2
shear modes	28.7 (1.0) E_{2g} [R]	6.4 (0) E'' [R]	7.2 (0.0001) E_g [R]	9.1 (0) E'' [R]	10.9 (0.03) E_g [R]	15.1 (0) E'' [R]	20.7 (1.0) E_g [R]
		12.1 (0.006) E' [IR+R]	14.0 (0) E_u [IR]	16.9 (0.04) E' [IR+R]	20.2 (0) E_u [IR]	25.5 (1.0) E' [IR+R]	
		17.5 (0) E'' [R]	20.1 (0.04) E_g [R]	23.0 (0) E'' [R]	26.6 (1.0) E_g [R]		
		22.1 (0.09) E' [IR+R]	24.6 (0) E_u [IR]	27.3 (1.0) E' [IR+R]			
		25.6 (0) E'' [R]	27.6 (1.0) E_g [R]				
		28.0 (1.0) E' [IR+R]					
breathing modes	45.9 (0) B_{2g} [Ina]	8.8 (0.34) A'1 [R]	11.6 (0.33) A_{1g} [R]	14.6 (0.30) A'_1 [R]	17.6 (0.32) A_{1g} [R]	22.1 (0.49) A'_1 [R]	30.8 (0.36) A_{1g} [R]
		19.9 (0) A''_2 [IR]	22.9 (0) A_{2u} [IR]	26.8 (0) A''_2 [IR]	31.9 (0) A_{2u} [IR]	39.7 (0) A''_2 [IR]	
		28.1 (0.03) A'_1 [R]	32.2 (0.001) A_{1g} [R]	36.9 (0.021) A'_1 [R]	42.2 (0.012) A_{1g} [R]		
		35.6 (0) A''_2 [IR]	39.4 (0) A_{2u} [IR]	43.1 (0) A''_2 [IR]			
		41.0 (0.005) A'_1 [R]	44.1 (0.000) A_{1g} [R]				
		44.8 (0) A''_2 [IR]					

Table S6 LDA calculated Γ point phonon frequencies (cm^{-1}) and relative Raman intensities I_{xx} (shown in parentheses) of the interlayer vibration modes in ABC stacked 2-7TL and bulk $MoSe_2$. The notations follow Table S4.

LDA calculation	ABC stacked few trilayer $MoSe_2$						
Thickness	bulk	7	6	5	4	3	2
shear modes		6.6 (0.18) E [IR+R]	7.6 (0.20) E [IR+R]	9.5 (0.11) E [IR+R]	10.4 (0.14) E [IR+R]	14.4 (0.23) E [IR+R]	20.7 (0.41) E [IR+R]
		12.4 (1e-9) E [IR+R]	14.2 (1e-8) E [IR+R]	17.1 (1e-6) E [IR+R]	20.4 (5e-6) E [IR+R]	25.0 (1e-5) E [IR+R]	
		17.9 (0.01) E [IR+R]	20.4 (0.016) E [IR+R]	23.4 (0.007) E [IR+R]	26.8 (0.006) E [IR+R]		
		22.5 (2e-6) E [IR+R]	25.1 (3e-6) E [IR+R]	27.6 (2e-6) E [IR+R]			
		26.1 (0.002) E [IR+R]	27.9 (0.001) E [IR+R]				
		28.3 (1e-6) E [IR+R]					
breathing modes		9.2 (1.0) A_1 [IR+R]	11.2 (1.0) A_1 [IR+R]	12.4 (1.0) A_1 [IR+R]	16.6 (1.0) A_1 [IR+R]	23.0 (1.0) A_1 [IR+R]	30.7 (1.0) A_1 [IR+R]
		18.7 (1e-6) A_1 [IR+R]	21.4 (1e-6) A_1 [IR+R]	25.4 (1e-6) A_1 [IR+R]	30.9 (1e-6) A_1 [IR+R]	37.3 (6e-7) A_1 [IR+R]	
		26.9 (0.08) A_1 [IR+R]	30.5 (0.08) A_1 [IR+R]	35.3 (0.05) A_1 [IR+R]	40.3 (0.03) A_1 [IR+R]		
		33.8 (2e-7) A_1 [IR+R]	37.6 (4e-7) A_1 [IR+R]	41.4 (6e-7) A_1 [IR+R]			
		39.2 (0.012) A_1 [IR+R]	41.8 (0.006) A_1 [IR+R]				
		42.3 (2e-7) A_1 [IR+R]					

Table S7 LDA calculated Γ point phonon frequencies (cm^{-1}) and relative Raman intensities I_{xx} (shown in parentheses) of the interlayer vibration modes in AB stacked 2-7TL and bulk WSe_2. The notations follow Table S1.

LDA calculation	AB stacked few trilayer WSe_2						
Thickness	bulk	7	6	5	4	3	2
shear modes	24.6 (1.0) E_{2g} [R]	5.4 (0) E" [R]	6.3 (0.004) E_g [R]	7.5 (0) E" [R]	9.4 (0.08) E_g [R]	12.4 (0) E" [R]	17.8 (1.0) E_g [R]
		10.6 (0.006) E' [IR+R]	12.2 (0) E_u [IR]	14.4 (0.03) E' [IR+R]	17.4 (0) E_u [IR]	21.6 (1.0) E' [IR+R]	
		15.2 (0) E" [R]	17.4 (0.09) E_g [R]	19.9 (0) E" [R]	23.0 (1.0) E_g [R]		
		19.2 (0.07) E' [IR+R]	21.3 (0) E_u [IR]	23.5 (1.0) E' [IR+R]			
		22.1 (0) E" [R]	23.9 (1.0) E_g [R]				
		24.0 (1.0) E' [IR+R]					
breathing modes	37.7 (0) B_{2g} [Ina]	8.5 (0.40) A'1 [R]	9.9 (0.57) A_{1g} [R]	11.8 (0.41) A'_1 [R]	14.8 (1.0) A_{1g} [R]	19.4 (0.73) A'_1 [R]	27.7(0.40) A_{1g} [R]
		16.5 (0) A''_2 [IR]	19.1 (0) A_{2u} [IR]	22.4 (0) A''_2 [IR]	27.0 (0) A_{2u} [IR]	33.4 (0) A''_2 [IR]	
		23.7 (0.04) A'_1 [R]	27.0 (0.05) A_{1g} [R]	30.7 (0.03) A'_1 [R]	35.5 (0.04) A_{1g} [R]		
		29.6 (0) A''_2 [IR]	33.0 (0) A_{2u} [IR]	36.1 (0) A''_2 [IR]			
		34.1 (0.007) A'_1 [R]	36.7 (0.005) A_{1g} [R]				
		36.9 (0) A''_2 [IR]					

Table S8 LDA calculated Γ point phonon frequencies (cm^{-1}) and relative Raman intensities I_{xx} (shown in parentheses) of the interlayer vibration modes in ABC stacked 2-7TL and bulk WSe_2. The notations follow Table S4.

LDA calculation	ABC stacked few trilayer WSe_2						
Thickness	bulk	7	6	5	4	3	2
shear modes	19.8 (1e-9) E [IR+R]	5.0 (0.29) E [IR+R]	5.9 (0.29) E [IR+R]	7.0 (0.29) E [IR+R]	8.7 (0.29) E [IR+R]	11.5 (0.29) E [IR+R]	16.5 (0.29) E [IR+R]
	19.8 (1e-9) E [IR+R]	9.9 (1e-9) E [IR+R]	11.4 (1e-8) E [IR+R]	13.4 (1e-6) E [IR+R]	16.3 (5e-6) E [IR+R]	19.9 (1e-5) E [IR+R]	
		14.2 (0.02) E [IR+R]	16.2 (0.02) E [IR+R]	18.5 (0.02) E [IR+R]	21.3 (0.009) E [IR+R]		
		17.9 (2e-6) E [IR+R]	19.8 (3e-6) E [IR+R]	21.8 (2e-6) E [IR+R]			
		20.6 (0.004) E [IR+R]	22.1 (0.002) E [IR+R]				
		22.3 (1e-6) E [IR+R]					
breathing modes	30.6 (1e-9) A_1 [IR+R]	7.9 (1.0) A_1 [IR+R]	9.2 (1.0) A_1 [IR+R]	10.9 (1.0) A_1 [IR+R]	13.7 (1.0) A_1 [IR+R]	17.9 (1.0) A_1 [IR+R]	25.7 (1.0) A_1 [IR+R]
	30.6 (1e-9) A_1 [IR+R]	15.4 (1e-6) A_1 [IR+R]	17.8 (1e-6) A_1 [IR+R]	20.9 (1e-6) A_1 [IR+R]	25.3 (1e-6) A_1 [IR+R]	31.1 (6e-7) A_1 [IR+R]	
		22.2 (0.09) A_1 [IR+R]	25.2 (0.08) A_1 [IR+R]	28.8 (0.06) A_1 [IR+R]	33.0 (0.03) A_1 [IR+R]		
		27.8 (2e-7) A_1 [IR+R]	30.8 (4e-7) A_1 [IR+R]	33.7 (6e-7) A_1 [IR+R]			
		31.9 (0.02) A_1 [IR+R]	34.2 (0.007) A_1 [IR+R]				
		34.5 (2e-7) A_1 [IR+R]					

Table S9 LDA calculated Γ point phonon frequencies (cm^{-1}) and relative Raman intensities I_{xx} (shown in parentheses) of the interlayer vibration modes in ABC stacked 2-5 QL and bulk Bi_2Se_3. The bulk Bi_2Se_3 is calculated with the hexagonal representation with 15 atoms in the unit cell. The ABC stacked multi- QL Bi_2Se_3 has the same symmetry as the ABC staking FLG, the notations follow Table S1.

LDA calculation	ABC stacked few quintuple layer Bi_2Se_3				
Thickness	bulk	5	4	3	2
shear modes	18.3 (1e-15) E_g [R]	7.2 (0.31) E_g [R]	7.8 (0.15) E_g [R]	11.1 (1.0) E_g [R]	15.0 (0.60) E_g [R]
	18.3 (0) E_u [IR]	12.8 (0) E_u [IR]	15.9 (0) E_u [IR]	16.9 (0) E_u [IR]	
		16.1 (0.001) E_g [R]	19.1 (0.01) E_g [R]		
		19.3 (0) E_u [IR]			
breathing modes	27.2 (1e-10) A_{1g} [R]	8.5 (1.0) A_{1g} [R]	11.2 (1.0) A_{1g} [R]	16.1 (0.69) A_{1g} [R]	22.0 (1.0) A_{1g} [R]
	27.2 (0) A_{2u} [IR]	18.7 (0) A_{2u} [IR]	21.5 (0) A_{2u} [IR]	29.7 (0) A_{2u} [IR]	
		26.5 (0.08) A_{1g} [R]	32.1 (0.10) A_{1g} [R]		
		32.5 (0) A_{2u} [IR]			

Table S10. Force constants (in 10^{19} N/m^3) and the derived elastic constants (in GPa) in AB and ABC stacked few layer graphene, $MoSe_2$, MoS_2, WSe_2 and Bi_2Se_3, obtained by fitting the DFT results to a linear chain model. The elastic constants are estimated from the in-plane and out-of-plane force constants by $C_{44}=k_x d$ and $C_{33}=k_z d$, where d is the distance between adjacent layers.

		k_x	k_z	C_{44}	C_{33}
C	C_AB	1.40	9.63	4.65	32.0
	C_ABC	1.46	9.63	4.85	32.0
	Exp. (AB)	1.28[a]	11.3[c]	4.3[a], 5.0[b]	37.5[c], 38.7[b]
$MoSe_2$	$MoSe_2$_AB	3.39	7.50	20.5	45.3
	$MoSe_2$_ABC	3.39	7.50	20.5	45.3
	Exp. (AB)	3.97		24.0	
	Exp. (ABC)	3.97		24.0	
MoS_2	MoS_2_AB	3.51	9.26	21.2	55.9
	MoS_2_ABC	4.1	8.24	24.8	49.8
	Exp. (AB)	2.72	8.62	18.9[d], 16.4	52[d] , 52
WSe_2	WSe_2_AB	3.41	8.38	21.7	53.4
	WSe_2_ABC	2.88	6.96	18.3	44.3
	Exp. (AB)	3.07	8.63	18.6	52.1
Bi_2Se_3	Bi_2Se_3_ABC	2.98	6.11	27.7	56.7
	Exp. (ABC)	2.27[e]	5.26 [e]	26.4 [e]	50.2 [e]

[a] Ref.[1], [b] Ref.[7] (measured from bulk graphite), [c] estimated from Ref.[3], [d] Ref.[8] (measured from bulk MoS_2), [e]Ref.[9]

Reference:

1. Tan PH*, et al.* The shear mode of multilayer graphene. *Nature materials* **11**, 294-300 (2012).

2. Boschetto D*, et al.* Real-Time Observation of Interlayer Vibrations in Bilayer and Few-Layer Graphene. *Nano Letters* **13**, 4620-4623 (2013).

3. Lui CH, Heinz TF. Measurement of layer breathing mode vibrations in few-layer graphene. *Physical Review B* **87**, 121404 (2013).

4. Lui CH, Li Z, Chen Z, Klimov PV, Brus LE, Heinz TF. Imaging Stacking Order in Few-Layer Graphene. *Nano Letters* **11**, 164-169 (2011).

5. Cong C*, et al.* Raman Characterization of ABA- and ABC-Stacked Trilayer Graphene. *ACS Nano* **5**, 8760-8768 (2011).

6. Luo X*, et al.* Effects of lower symmetry and dimensionality on Raman spectra in two-dimensional WSe 2. *Physical Review B* **88**, 195313 (2013).

7. Bosak A, Krisch M, Mohr M, Maultzsch J, Thomsen C. Elasticity of single-crystalline graphite: inelastic X-ray scattering study. *Physical Review B* **75**, 153408 (2007).

8. Feldman JL. Elastic constants of 2H-MoS2 and 2H-NbSe2 extracted from measured dispersion curves and linear compressibilities. *Journal of Physics and Chemistry of Solids* **37**, 1141-1144 (1976).

9. Zhao Y*, et al.* Interlayer vibrational modes in few-quintuple-layer Bi2Te3 and Bi2Se3 two-dimensional crystals: Raman spectroscopy and first-principles studies. *Physical Review B* **90**, 245428 (2014).
